# A Bayesian Network Analysis of the Probabilistic Relationships Between Various Obesity Phenotypes and Cardiovascular Disease Risk in Chinese Adults: Chinese Population-Based Observational Study

**DOI:** 10.2196/33026

**Published:** 2022-03-02

**Authors:** Simiao Tian, Mei Bi, Yanhong Bi, Xiaoyu Che, Yazhuo Liu

**Affiliations:** 1 Department of Research Affiliated Zhongshan Hospital of Dalian University Dalian China; 2 Department of Clinical Nutrition and Metabolism Affiliated Zhongshan Hospital of Dalian University Dalian China

**Keywords:** Bayesian network, metabolic health, obesity, cardiovascular disease risk

## Abstract

**Background:**

Cardiovascular disease (CVD) risk among individuals with different BMI levels might depend on their metabolic health. The extent to which metabolic health status and BMI affect CVD risk, either directly or through a mediator, in the Chinese population remains unclear.

**Objective:**

In this study, the Bayesian network (BN) perspective is adopted to characterize the multivariable probabilistic connections between CVD risk and metabolic health and obesity status and identify potential factors that influence these relationships among Chinese adults.

**Methods:**

The study population comprised 6276 Chinese adults aged 30 to 74 years who participated in the China Health and Nutrition Survey 2009. BMI was used to categorize participants as normal weight, overweight, or obese, and metabolic health was defined by the Adult Treatment Panel-3 criteria. Participants were categorized into 6 phenotypes according to their metabolic health and BMI categorization. The 10-year risk of CVD was determined using the Framingham Risk Score. BN modeling was used to identify the network structure of the variables and compute the conditional probability of CVD risk for the different metabolic obesity phenotypes with the given structure.

**Results:**

Of 6276 participants, 64.67% (n=4059), 20.37% (n=1279), and 14.95% (n=938) had a low, moderate, and high 10-year CVD risk. An averaged BN with a stable network structure was constructed by learning 300 bootstrapped networks from the data. Using BN reasoning, the conditional probability of high CVD risk increased as age progressed. The conditional probability of high CVD risk was 0.43% (95% CI 0.2%-0.87%) for the 30 to 40 years age group, 2.25% (95% CI 1.75%-2.88%) for the 40 to 50 years age group, 16.13% (95% CI 14.86%-17.5%) for the 50 to 60 years age group, and 52.02% (95% CI 47.62%-56.38%) for those aged ≥70 years. When metabolic health and BMI categories were instantiated to their different statuses, the conditional probability of high CVD risk increased from 7.01% (95% CI 6.27%-7.83%) for participants who were metabolically healthy normal weight to 10.47% (95% CI 7.63%-14.18%) for their metabolically healthy obese (MHO) counterparts and up to 21.74% and 34.48% among participants who were metabolically unhealthy normal weight and metabolically unhealthy obese (MUO), respectively. Sex was a significant modifier of the conditional probability distribution of metabolic obesity phenotypes and high CVD risk, with a conditional probability of high CVD risk of only 2.02% and 22.7% among MHO and MUO women, respectively, compared with 21.92% and 48.21% for their male MHO and MUO counterparts, respectively.

**Conclusions:**

BN modeling was applied to investigate the relationship between CVD risk and metabolic health and obesity phenotypes in Chinese adults. The results suggest that both metabolic health and obesity status are important for CVD prevention; closer attention should be paid to BMI and metabolic status changes over time.

## Introduction

### Background

Cardiovascular disease (CVD) is becoming a leading cause of mortality, disability, and rising health care costs worldwide [1,2]. The worldwide prevalence of CVD doubled from 271 million in 1990 to 523 million in 2019 [1], and recent epidemiological studies indicate that CVD accounts for >40% of deaths in the general Chinese population [3]. Despite significant efforts directed toward CVD prevention and control at the individual level and public health level, there has still been a clear increase in deaths because of CVD in China over the past 2 decades, from 2.51 million in 1990 to 3.97 million in 2016, as well as a doubled prevalence from 1990 to 2016 [2,3].

Obesity is recognized as the primary cause of many chronic diseases. It is also an established risk factor for CVDs, including coronary disease [4], myocardial infarction [5], and ischemic heart disease [6]. Previous cohort studies have reported a causal relationship between obesity and increased risk of CVD mortality [7]. Along with obesity, metabolic syndrome (MetS), which is a cluster of interrelated metabolic abnormalities, including increased blood pressure (BP), hyperglycemia, central adiposity, insulin resistance, and dyslipidemia, is another well-established determinant of CVD and mortality [8]. However, there is heterogeneity in body fat distribution and metabolic factors among individuals with obesity, and it has been reported that a subgroup of people with obesity possesses a favorable cardiometabolic profile; these individuals are referred to as people who are metabolically healthy (MH) obese. They may not be at increased risk of several health outcomes, including CVD [9], and may even confer a protective effect on all-cause mortality if accompanied by a healthy metabolism [10]. Together, the findings of these studies highlight the need to take metabolic health and obesity status into account in CVD-related studies.

In general, any condition or disease that affects the heart, its vessels, and the blood circulatory system [11] or is associated with conditions such as chronic heart failure (HF), congenital heart disease, rhythm disorders, and subclinical atherosclerosis [12] can be related to CVD. In addition to the main risk factors, recently published studies have highlighted the important role of other factors such as infection, inflammatory conditions, and chronic diseases in CVD development [13]. CVD is a multicausal disease and presents a clear heterogeneity in terms of prevalence and mortality among various subgroups that differ in their demographic characteristics; therefore, sex, age, smoking, high cholesterol, hypertension, and diabetes should be taken into account in CVD studies [14], together with metabolic health and BMI levels. Modeling multiple correlated factors when assessing CVD risk can be computationally challenging and requires new statistical approaches. Standard regression modeling requires independence among covariates and cannot disentangle the interrelationships or interactions that form complex networks of relationships. Bayesian networks (BNs) are powerful probabilistic graphical models that enable the description of conditional dependencies and reasoning among a set of variables, permitting a comprehensive investigation of interrelationships among multiple correlated variables and identification of potential causality [15]. The generated BN model can be used for dynamic qualitative and quantitative reasoning, where the probability of all variables changes by updating the state of one variable, revealing inferences between the depicted variables [16]. Recently, BNs have been extensively used in health science and epidemiology, particularly in CVD research in areas such as diagnosis, risk assessment, and disease prediction [17-19]. To our knowledge, few studies have examined the interrelationships between metabolic health status, BMI, and CVD risk in conjunction with demographic factors, biomarkers, and chronic health outcomes in the Chinese population.

### Objective

This study aims to fill this gap in knowledge by introducing BN modeling and evaluating the multivariable probabilistic connections among metabolic health, obesity, and CVD risk in a population-level study of Chinese adults. In addition, this study aims to identify factors that directly and indirectly influence these relationships.

## Methods

### Study Population

The participants in this study were recruited from the China Health and Nutrition Survey (CHNS), which is an ongoing longitudinal survey designed to examine the effects of health and nutrition at the population level. A detailed description of the CHNS, such as the multistage sampling design and data collection methods, has been provided elsewhere [20], and this study uses a cohort that has been previously described [21]. Briefly, the participants included in this study were obtained from the 2009 CHNS wave (N=11,929). The participants voluntarily participated in health interviews and examinations, answered the general sociodemographic questions, and completed an in-depth health questionnaire. Data were collected via household interviews.

### Ethics Approval and Consent to Participate

The CHNS study was approved by the institutional review committees of the University of North Carolina at Chapel Hill, the National Institute of Nutrition and Food Safety, Chinese Centers for Disease Control and Prevention, the China–Japan Friendship Hospital, and the Ministry of Health (R01-HD30880, DK056350, and R01-HD38700). All participants provided written informed consent. All methods were performed in accordance with the relevant guidelines and regulations.

### Data Collection and Measurements

Face-to-face interviews were conducted by well-trained personnel using self-administered and standardized questionnaires. The interviews were used to collect information on participants’ demographic characteristics (age, sex, marital status, and education level), behavioral factors (smoking status, drinking status, and physical activity), medication use, and self-reported family history.

All participants underwent a physical examination performed by well-trained examiners, following standardized procedures. Body weight and height were measured to the nearest 0.1 kg and 0.1 cm, respectively, with the participants wearing light clothing and no shoes. Waist circumference was measured to the nearest 0.1 cm at the midpoint between the bottom of the rib cage and the top of the iliac crest following exhalation. BMI was calculated as weight (kg) divided by the square of height (meters). Systolic BP (SBP) and diastolic BP (DBP) were measured using a standardized mercury sphygmomanometer on the participant’s right arm. BP measurements were performed in triplicate after 10 minutes of seated rest, and the mean of the 3 measurements was used in the analyses.

The participants were required to fast overnight before blood collection. Fasting blood samples were obtained the following morning using a standardized process and were then analyzed in a national central clinical laboratory in Beijing. Plasma and serum samples were frozen and stored at −86 °C for later laboratory analyses. Serum levels of fasting plasma glucose (FPG), total cholesterol (TC), low-density lipoprotein cholesterol, high-density lipoprotein cholesterol (HDL-C), triglyceride (TG), uric acid (UA), and other routine blood biochemical indices were measured using a biochemical autoanalyzer. Details of all laboratory analyses and measurements can be found elsewhere [20]. Homeostasis model assessment of insulin resistance (HOMA-IR) was calculated using the following formula:

HOMA-IR=fasting insulin (microinternational units per milliliter)×FPG (millimoles per liter)/22.5 **(1)**

Fasting serum was used to derive the serum creatinine concentration (mg/dL). The estimated glomerular filtration rate (eGFR) was calculated using the Chronic Kidney Disease Epidemiology equations combined with the serum creatinine equation. The robust performance of serum creatinine–based equations has been validated in the Chinese population [22].

In this study, according to the criteria recommended by the US Joint National Committee and Chinese guidelines [23,24], hypertension was defined as an SBP ≥140 mm Hg, a DBP ≥90 mm Hg, and/or the self-reported use of antihypertensive medication. Diabetes was defined as FPG ≥7.0 mmol/L or treatment for diabetes. On the basis of the National Cholesterol Education Project guidelines [25], dyslipidemia was defined as low-density lipoprotein cholesterol ≥4.14 mmol/L, HDL-C ≤1.036 mmol/L, and TGs ≥2.26 mmol/L. Hyperuricemia was defined as serum UA ≥420 µmol/L in men and ≥360 µmol/L in women [26].

### Assessment of 10-Year Risk of CVD

The Framingham Risk Score (FRS) was used to estimate the 10-year probability of a CVD event (coronary heart disease, cerebrovascular event, peripheral artery disease, or HF). The FRS was developed by D’Agostino et al [27] using a sex-specific multivariable risk factor algorithm and has been validated in American, Canadian, European, and Asian populations [28-31], as well as in Chinese participants [32]. It is used in primary care to assess overall cardiovascular risk among participants who are asymptomatic at baseline and are aged 30 to 74 years, providing clinicians with quantitative information to aid in the targeted lowering of risk factors [33]. As per the conditions of the FRS algorithm, participants s aged <30 years or >74 years, as well as those with incomplete data with respect to the anthropometric measures and blood sampling, were excluded. As a result, a total of 6276 individuals (2895, 46.13%) men and (3381, 53.87%) women were enrolled in this study.

The raw FRS score was calculated for each participant based on the individual’s sex, age, TC, smoking status, HDL-C, SBP (treatment for hypertension and SB*P* value), and diabetes status, together with their associated proper β coefficient value from the proportional hazard regression [27]. The 10-year risk factor was then derived as a percentage by gender. In addition, the 10-year CVD risk was categorized as low (FRS <10%), moderate (10%-19%), or high (≥20%) according to previous recommendations [13,28].

### Definitions of Obesity, Metabolic Health, and Metabolic Obesity Phenotypes

Overweight and obesity were defined as BMI≥24 kg/m² and ≥28 kg/m², respectively, using the criteria for Chinese adults [34,35]. Each participant was then categorized into one of three BMI groups: normal weight (BMI 18.5-23.9 kg/m²), overweight (BMI 24.0-27.9 kg/m²), and obese (BMI ≥28.0 kg/m²).

The metabolic health status of each participant was defined based on the Adult Treatment Panel-3 definition of MetS [36]. Participants who met ≥2 of the following four criteria were considered metabolically unhealthy (MU): (1) hypertension (SBP/DBP ≥130/85 mm Hg or use of antihypertensive drugs), (2) hypertriglyceridemia (TG ≥1.7 mmol/L or use of lipid-lowering drugs), (3) hyperglycemia (FPG ≥5.6 mmol/L or use of medications for diabetes), and (4) reduced HDL-C (HDL-C <1.04 mmol/L for men and <1.3 mmol/L for women). The waist circumference criterion was not used because of collinearity with BMI.

The above definition was used together with the BMI categories to classify the study participants into one of six following metabolic obesity phenotypes: participants who are MH participants with normal weight, participants who are MH and overweight, participants who are MH and obese (MH normal weight [MHNW], MH overweight [MHOW], and MH obese [MHO], respectively), participants who are MU with normal weight, participants who are MU and overweight, and participants who are MU and obese (MU normal weight [MUNW], MU overweight [MUOW], and MU obese [MUO], respectively).

### BN Modeling

A BN is a probabilistic graphical model that represents a set of random variables *X* = {X_i_,...,X_n_} as nodes and their conditional dependencies as edges through a directed acyclic graph (DAG) [16]. A BN can be fully specified by a pair (G, P), in which G=(V, A) is a DAG comprising nodes (denoted by V) and directed edges (denoted by A) and P is a joint probability distribution. Specifically, if there is an edge from node X_i_ to node X_j_, X_i_ is then termed the parent and X_j,_ the child, and the direction of the edge indicates a statistical dependence between the corresponding variables. The joint distribution P can be written as the product of the local conditional probability of each node X_i_, given its parent variables in graph G, as follows:



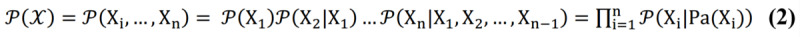



Pa(X_i_) are the parents of X_i_ in the BN, and P(X) reflects the properties of the BN.

The BN models were built and reviewed through an iterative 2-stage process, including a stepwise manual construction process and a data-driven approach [37,38]. First, a manual construction approach was used to explore different network structures by including various sets of potential risk factors or variables consecutively. The selection of the variable nodes was based on prior expert knowledge and a systematic review of the literature, which has been shown to improve BN structure learning processes and to avoid excessive complexity of the network structure by the inclusion of too many nodes [39]. During this first stage, prior knowledge can be included in the model as blacklist and whitelist arcs. Specifically, the directions between certain variables were restricted by using a layering approach [40]. For instance, the variables metabolic health status and BMI were allowed to be directed to FRS categories, and this setting ensured that information was embedded in the direction of causality for the effect of different obesity phenotypes (whitelist), and the FRS categories were not permitted to influence age (blacklist), as we were interested in understanding the age-related pathways that explain 10-year CVD risk as an outcome.

Second, a data-driven approach using different structure learning algorithms was adopted to further improve the BN. On the basis of prespecified simulations and comparison among score-based, constraint-based, and hybrid structure learning approaches, the tabu-search algorithm [41] was used for graphical structure learning along with the Bayesian information criterion score [42] to achieve high quality of the network structure. The stabilities of the arcs in the network were examined from 300 bootstrapped networks, and the arc strengths (between 0 and 1) were estimated by averaging the probability of the arcs presenting in these bootstrap-resampled network structures [21,43]. The final BN model was obtained by using the structure and directions of arcs from the averaged network and was then further used to query the conditional probability distributions (Bayesian reasoning) with a specific value or evidence provided.

### Statistical Analyses

Continuous variables were presented as medians or means with SD according to its assumption of normality from the Kolmogorov–Smirnov test, and categorical variables were presented as numerical variables with the corresponding proportions as functions of metabolic health status and BMI. Comparison of the different obesity phenotypes was performed using 1-way analysis of variance or the Kruskal-Wallis test for continuous variables, where appropriate, or the chi-square test for categorical variables. *P* values for trend were computed using Pearson for continuous variables and the Mantel–Haenszel chi-square test for categorical variables. All statistical analyses were performed using R (version 3.2.2; R Foundation for Statistical Computing) software [44], and *P*<.05 was considered statistically significant. The bnlearn package in the R software environment was used for BN modeling analysis [43], including network structure learning, parameter estimation, network arc stabilities, conditional probability queries in the finalized network, and visualization. The data and code for full analysis can be obtained by reasonable request from the corresponding author.

## Results

### Characteristics of the Sample

The characteristics of the sample, stratified by BMI and metabolic health status, are shown in Table 1. Among the 3881 participants without MetS, 2548 (65.65%) had a normal BMI, and 198 (5.1%) had MHO. Among 2395 participants with MU profiles, 955 (39.87%), 981 (40.96%), and 459 (19.16%) were classified into the MUNW, MUOW, and MUO groups, respectively. Within the same BMI levels, the MU groups more commonly exhibited greater waist and hip circumference measurements, along with elevated BP, TC, TG, and UA, and lower levels of HDL-C than the MH groups. In particular, glucose biomarkers, including FPG, HOMA-IR, and Hemoglobin A_1c_, were higher in the participants who were MU than in their healthy counterparts (Table 1). The distributions of age groups, sex, smoking, and alcohol drinking status also differed among the 6 groups (*P*<.001*, P=*.002*, P<*.001*,* and *P=*.01*,* respectively). Participants who were MHO were more often women and were more commonly nonsmokers and nondrinkers compared with their obese counterparts with MetS (MUO), whereas a more unfavorable risk profile was seen in the MUO group than in the MHO group. Among the 955 participants in the MUNW group, 419 (43.9%) had hypertension, 112 (11.7%) had diabetes, and 545 (57.1%) had dyslipidemia. Among the 459 participants in the MUO group, the prevalence of the abovementioned cardiometabolic disorders was 279 (60.8%), 74 (16.1%), and 338 (73.6%), respectively.

**Table 1:** Characteristics of study sample based on combinations of BMI and metabolic health defined by Adult Treatment Panel-3 criteria (N=6276).

Characteristics		MHNW^a^ (n=2548)	MHOW^b^ (n=1135)	MHO^c^ (n=198)	MUNW^d^ (n=955)	MUOW^e^ (n=981)	MUO^f^ (n=459)	*P* value^g^	*P* value for trend^h^
Age (years), mean (SD)		49.53 (11.21)	50.43 (10.66)	49.68 (10.88)	54.66 (10.77)	53.15 (10.30)	52.36 (10.68)	<.001	<.001
Sex (male), n (%)		1188 (46.6)	507 (44.7)	71 (35.9)	432 (45.2)	496 (50.6)	201 (43.8)	.002	.59
Smoker, n (%)		859 (33.7)	314 (27.7)	37 (18.7)	315 (33)	341 (34.8)	125 (27.2)	<.001	.49
Alcohol drinker, n (%)		882 (34.6)	398 (35.1)	59 (29.8)	290 (30.4)	368 (37.5)	144 (31.4)	.01	.52
Weight (kg), mean (SD)		55.72 (6.83)	66.66 (7.14)	76.08 (9.27)	56.99 (7.18)	67.73 (7.82)	78.42 (9.45)	<.001	<.001
Height (cm), mean (SD)		160.86 (8.12)	161.25 (8.04)	160.10 (8.92)	160.70 (8.50)	161.83 (8.57)	161.00 (8.61)	.01	.12
BMI (kg/m^2^), mean (SD)		21.48 (1.44)	25.58 (1.10)	29.59 (1.51)	22.00 (1.40)	25.79 (1.12)	30.18 (1.92)	<.001	<.001
Waist circumference (cm), mean (SD)		77.76 (7.12)	87.30 (6.82)	95.40 (8.25)	80.70 (7.43)	89.06 (6.72)	98.27 (7.50)	<.001	<.001
Hip circumference (cm), mean (SD)		91.16 (5.47)	98.27 (5.75)	105.01 (6.14)	92.16 (6.24)	98.59 (5.39)	106.07 (6.15)	<.001	<.001
HDL-C^i^ (mmol/L), mean (SD)		1.58 (0.49)	1.46 (0.34)	1.47 (0.34)	1.31 (0.39)	1.20 (0.33)	1.18 (0.60)	<.001	<.001
LDL-C^j^ (mmol/L), mean (SD)		2.93 (0.91)	3.16 (0.84)	3.27 (0.88)	3.01 (1.07)	3.10 (1.05)	3.13 (1.23)	<.001	<.001
DBP^k^ (mm Hg), mean (SD)		76.98 (9.78)	80.54 (10.45)	83.17 (9.92)	84.06 (11.11)	86.21 (10.44)	89.75 (12.11)	<.001	<.001
SBP^l^ (mm Hg), mean (SD)		118.08 (15.30)	123.11 (16.81)	127.00 (14.80)	130.66 (18.46)	133.19 (18.00)	137.72 (20.37)	<.001	<.001
FPG^m^ (mmol/L), mean (SD)		4.94 (0.66)	5.04 (0.79)	5.13 (0.94)	5.91 (1.54)	5.92 (1.71)	6.11 (1.67)	<.001	<.001
TC^n^ (mmol/L), mean (SD)		4.71 (0.91)	4.90 (0.91)	5.00 (0.92)	5.04 (1.08)	5.21 (1.04)	5.22 (1.07)	<.001	<.001
TG^o^ (mmol/L), mean (SD)		1.09 (0.65)	1.27 (0.70)	1.25 (0.51)	2.33 (1.55)	2.82 (2.10)	2.96 (2.15)	<.001	<.001
Urea (mmol/L), mean (SD)		5.43 (1.63)	5.45 (1.45)	5.33 (1.19)	5.54 (1.45)	5.65 (1.48)	5.59 (1.47)	.001	<.001
Uric acid (µmol/L), mean (SD)		276.75 (82.64)	291.26 (84.42)	292.03 (75.75)	325.58 (106.51)	358.75 (131.02)	362.59 (115.99)	<.001	<.001
HOMA-IR^p^, mean (SD)		2.38 (3.15)	2.93 (4.40)	3.43 (2.74)	4.89 (11.37)	5.23 (7.93)	6.26 (6.58)	<.001	<.001
hsCRP^q^, mean (SD)		1.81 (5.24)	2.21 (5.25)	2.77 (4.40)	2.82 (10.08)	2.94 (5.66)	3.68 (5.22)	<.001	<.001
HbA_1C_^r^, mean (SD)		5.41 (0.53)	5.54 (0.53)	5.66 (0.69)	5.67 (0.94)	5.83 (0.94)	6.05 (1.02)	<.001	<.001
**Age groups, n (%)**									
	30-39	609 (23.9)	225 (19.8)	43 (21.7)	102 (10.7)	119 (12.1)	63 (14)	<.001	<.001
	40-49	738 (29)	349 (30.7)	63 (31.8)	220 (23)	252 (25.7)	139 (30.2)	<.001	<.001
	50-59	700 (27.5)	322 (28.4)	52 (26.3)	311 (32.6)	346 (35.3)	149 (32.5)	<.001	<.001
	60-69	390 (15.3)	194 (17.1)	33 (16.7)	246 (25.8)	212 (21.6)	78 (17)	<.001	<.001
	≥70	111 (4.4)	45 (4)	7 (3.5)	76 (8)	52 (5)	30 (7)	<.001	<.001
Hypertension, n (%)		379 (14.9)	251 (22.1)	70 (35.4)	419 (43.9)	511 (52.1)	279 (60.8)	<.001	<.001
Diabetes, n (%)		21 (0.8)	10 (0.9)	3 (1.5)	112 (11.7)	110 (11.2)	74 (16)	<.001	<.001
Dyslipidemia, n (%)		372 (14.6)	234 (20.6)	46 (23.2)	545 (57.1)	696 (71)	338 (73.6)	<.001	<.001
Hyperuricemia, n (%)		170 (6.7)	119 (10.5)	18 (9.1)	187 (19.6)	302 (30.8)	156 (34)	<.001	<.001

^a^MHNW: metabolically healthy normal weight.

^b^MHOW: metabolically healthy overweight.

^c^MHO: metabolically healthy obese.

^d^MUNW: metabolically unhealthy normal weight.

^e^MUOW: metabolically unhealthy overweight.

^f^MUO: metabolically unhealthy obese.

^g^The *P* value for overall comparison of the different obesity phenotypes.

^h^The *P* value for trend was computed from the Pearson test for continuous variables and the Mantel-Haenszel chi-square test for categorical variables.

^i^HDL-C: high-density lipoprotein cholesterol.

^j^LDL-C: low-density lipoprotein cholesterol.

^k^DBP: diastolic blood pressure.

^l^SBP: systolic blood pressure.

^m^FPG: fasting plasma glucose.

^n^TC: total cholesterol.

^o^TG: triglyceride.

^p^HOMA-IR: homeostatic model assessment of insulin resistance.

^q^hsCRP: high-sensitivity C-reactive protein.

^r^HbA_1c_: hemoglobin A_1c_.

### Distribution of FRS According to Obesity Phenotypes

The distribution of FRS according to metabolic health status and BMI is shown in Table 2. In general, the FRS increased with weight in both participants who were MH and participants who were MU. The average FRS among participants who were obese and with favorable metabolic profiles (MHO) was 7.54% (SD 7.91%), whereas the risk score doubled (14.16%, SD 13.01%) among participants who were MUNW and further increased to 15.98% (SD 14.42%) among participants who were MUO (Table 2).

With regard to FRS categories, among those with a MU profile, the proportion of participants with a high 10-year CVD risk ranged from 24% (229/955) in the MUNW group to 27.5% (125/459) in the MUO group. In addition, approximately half of all participants who were MU had a low 10-year CVD risk, with proportions of 50.2% (497/955), 46.1% (452/981), and 45.8% (210/459) among the MUNW, MUOW, and MUO groups, respectively. In contrast, among participants who were MH, a considerably higher proportion had a low 10-year CVD risk, and lower proportions had a high CVD risk regardless of BMI levels: 79.3% (157/198) of participants who were MHO had a low risk, whereas a high risk was only observed among 5.1% (10/198) of participants who were MHO. A similar pattern was noted in the MHNW and MHOW groups.

**Table 2:** Levels of and distribution of the Framingham Risk Score (FRS) among each obesity phenotype.

Distribution		MHNW^a^ (n=2548)	MHOW^b^ (n=1135)	MHO^c^ (n=198)	MUNW^d^ (n=955)	MUOW^e^ (n=981)	MUO^f^ (n=459)
FRS distribution, mean (SD)		7.43 (8.39)	8.54 (9.42)	7.54 (7.91)	14.16 (13.01)	15.63 (14.08)	15.98 (14.42)
**FRS categories, n (%)**							
	Low	1930 (75.75)	831 (73.22)	157 (79.3)	479 (50.2)	452 (46.1)	210 (45.8)
	Moderate	412 (16.17)	196 (17.27)	31 (15.7)	247 (25.9)	270 (27.5)	123 (26.8)
	High	206 (8.08)	108 (9.52)	10 (5.1)	229 (24)	259 (26.4)	126 (27.5)

^a^MHNW: metabolically healthy normal weight.

^b^MHOW: metabolically healthy overweight.

^c^MHO: metabolically healthy obese.

^d^MUNW: metabolically unhealthy normal weight.

^e^MUOW: metabolically unhealthy overweight.

^f^MUO: metabolically unhealthy obese.

### BN Development

**Figure 1:**
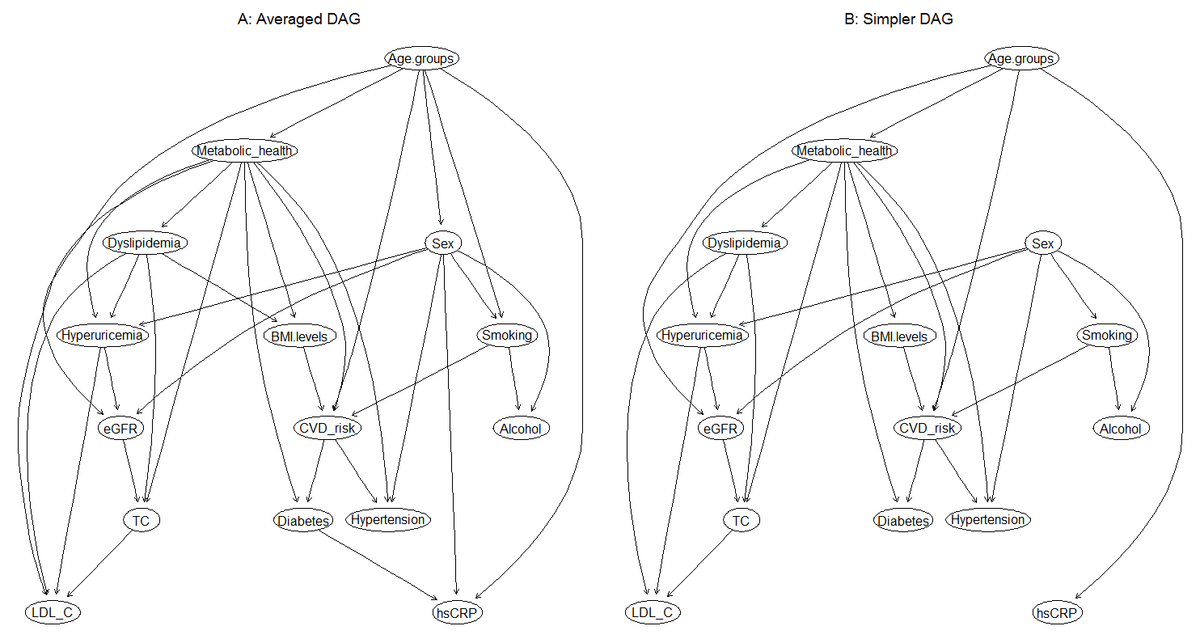
The directed acyclic graph (DAG) underlying the Bayesian network learned from 10-year cardiovascular disease (CVD) risk, the covariates, metabolic health, and obesity status. (A) Averaged DAG with strength of arcs >0.5; (B) simplified DAG derived from the averaged DAG after retaining arcs with a strength >0.85. eGFR: estimated glomerular filtration rate; hsCRP: high-sensitivity C-reactive protein; LDL-C: low-density lipoprotein cholesterol; TC: total cholesterol.

BN modeling was used to estimate the 10-year CVD risk among various obesity phenotypes. By using a whitelist and blacklist from prior expert knowledge, an averaged BN was constructed by learning 300 bootstrapped networks from the data and further retaining the arcs with an appearing frequency of at least 50%, as shown in Figure 1A. This BN model describes the interrelationships between demographic factors, behavioral factors, CVD risk factors, obesity phenotypes, and FRS categories, as well as the relationship between risk factors and demographic covariates. All the directions of the arcs seem to be well-established, and this could be attributed to the layering approach (whitelist and blacklist), which implements certain restrictions on the arc directions.

Then, a further examination was performed using the arc strength criteria to simplify the complexity of the BN, with little loss of information in the process. The final BN was obtained with an arc strength threshold >0.85 (ie, arcs appear with a frequency of at least 0.85 among the 300 bootstrapped networks), as depicted in Figure 1B (the direct comparison of arcs between the averaged BN and simplified BN is shown in Multimedia Appendix 1). All probability distributions are represented in the nodes, and the probabilistic dependencies are indicated by direct edges connecting the nodes. The connections between 10-year CVD risk, its risk factors, and obesity phenotypes were established by a complex network structure and assumed to be dependent, in which direct connections among metabolic health status, BMI level, age, smoking status, and CVD risk were identified (Figure 1B), together with an indirect link between sex and 10-year CVD risk through smoking status. In addition, metabolic health status and sex were directly connected with 7 and 5 covariates, respectively, indicating that they had the most children nodes, implying a sex-specific relationship. The interrelationships between various CVD risk factors are also presented in Figure 1B. For instance, eGFR was related to sex and age; hypertension and hyperuricemia were both associated with metabolic health status and sex; and TC was influenced by eGFR, dyslipidemia, and metabolic health status.

### BN Reasoning

**Figure 2:**
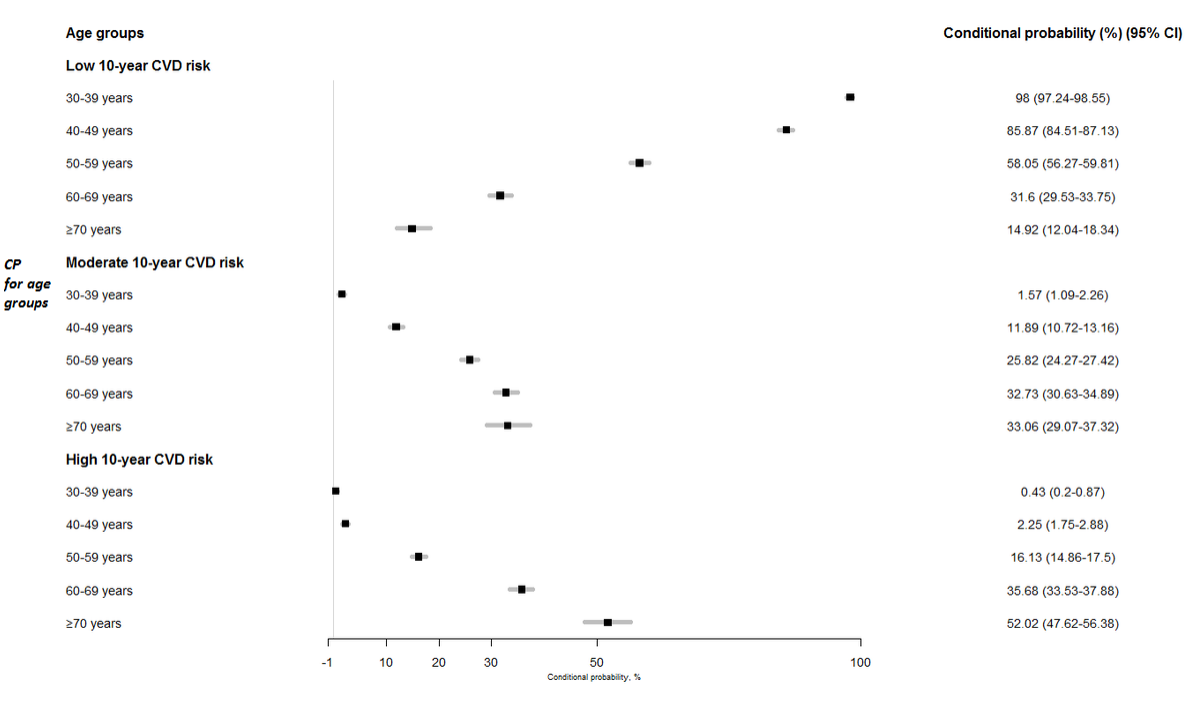
Conditional probabilities (in percentage) and 95% CIs of low, moderate, and high 10-year cardiovascular disease (CVD) risk in different age groups in Chinese adults.

BN reasoning was performed to estimate the conditional probabilities of the 10-year CVD risk, given various evidence from the well-built BN model. Since age had a significant modifying effect on the probability distribution of CVD, the variation in the conditional probabilities was estimated through the different age groups (Figure 2). The conditional probability of high CVD risk increased as age progressed, with a greater rise from 30 to 40 years (0.43%, 95% CI 0.2-0.87) to 40 to 50 years (2.25%, 95% CI 1.75-2.88) and then to 50 to 60 years (16.13%, 95% CI 14.86-17.5). Furthermore, more than half of the participants aged ≥70 years had a high CVD risk (52.02%, 95% CI 47.62-56.38). A similar pattern was also observed for moderate CVD risk among the different age groups, with a steady increase in the conditional probability observed with increasing age. In contrast, the probability of low CVD risk decreased from 98% (95% CI 97.24 to 98.55) for the 30 to 40 years age group to 58.05% (95% CI 56.27 to 59.81) for the 50 to 60 years age group to only 14.92% (95% CI 12.04 to 18.34) for those aged ≥70 years.

**Figure 3:**
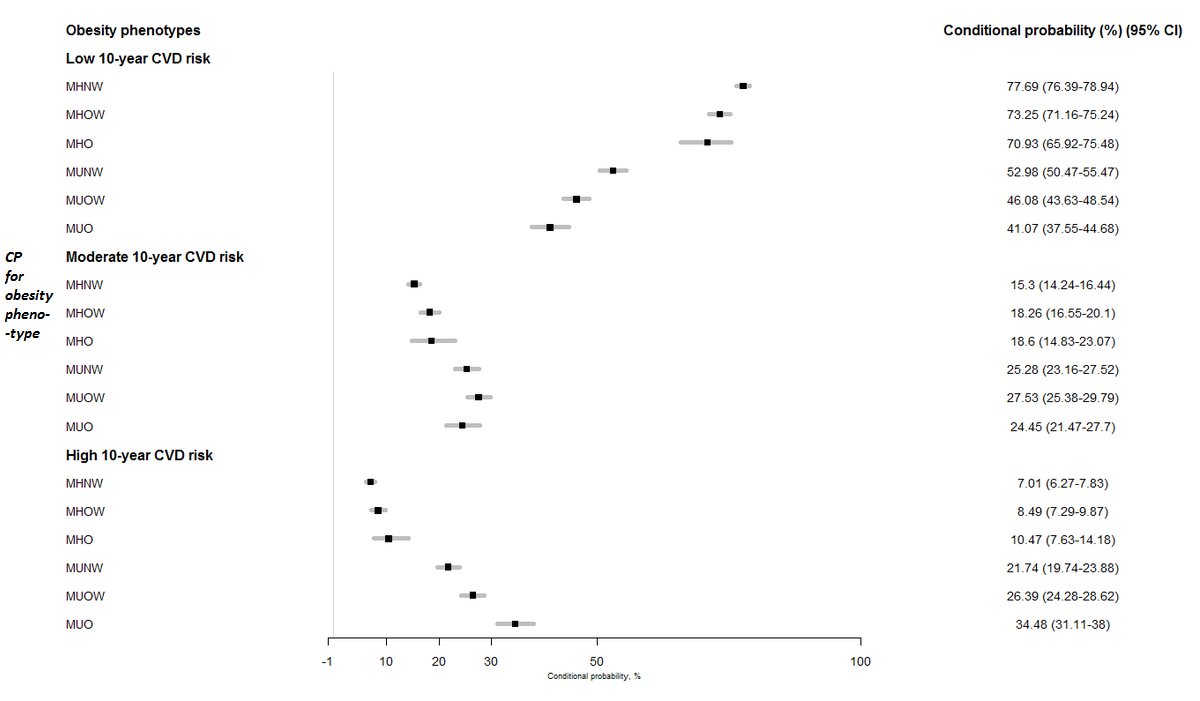
Conditional probabilities (in percentage) and 95% CIs of low, moderate, and high 10-year cardiovascular disease (CVD) risk in different obesity phenotypes. MHNW: metabolically healthy normal weight; MHO: metabolically healthy obese; MHOW: metabolically healthy overweight; MUNW: metabolically unhealthy normal weight; MUO: metabolically unhealthy obese; MUOW: metabolically unhealthy overweight.

The probability distributions of CVD risk were updated when providing evidence of BMI level and metabolic health status from the BN model (Figure 3). Among the participants with favorable metabolic health profiles, the conditional probability of having moderate or high CVD risk ranged from 15.3% and 7.01%, respectively, for the participants with normal weight (ie, MHNW) to 18.6% and 10.47%, respectively, for their obese counterparts (ie, MHO). In contrast, within the same BMI levels, the probabilities were 25.28% and 21.74%, respectively, for participants who were normal weight with a MU status (ie, MUNW) and further increased to 24.45% and 34.48%, respectively, among participants who were MUO. In addition, the conditional probabilities of low CVD risk exhibited a substantial decline from 77.69% to 52.98% when the metabolic health status of participants who were normal weight (ie, MHNW) became unfavorable (MUNW).

**Figure 4:**
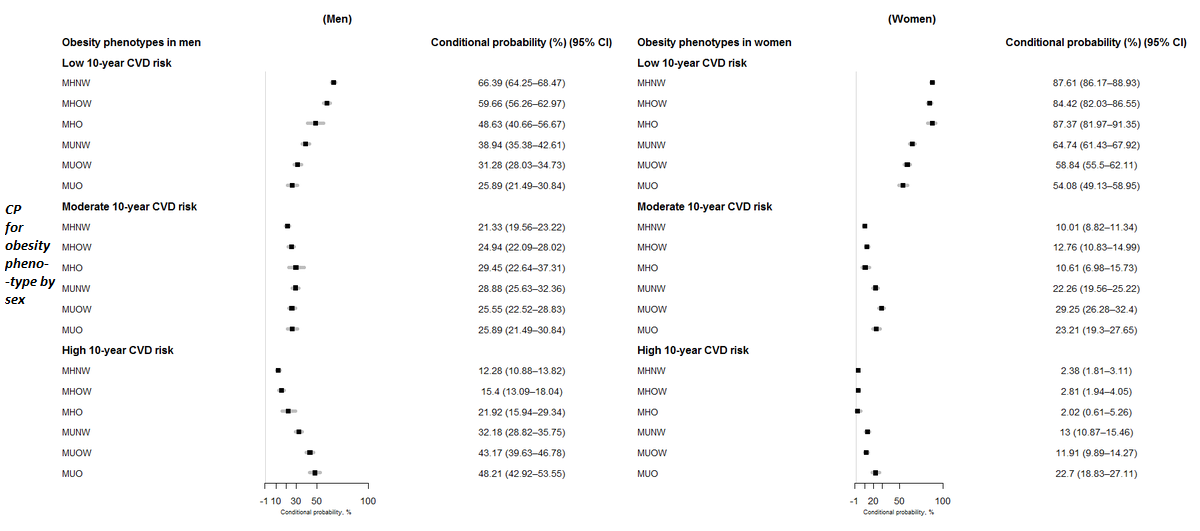
Conditional probabilities (in percentage) and 95% CIs of low, moderate, and high 10-year cardiovascular disease (CVD) risk in different obesity phenotypes by sex. MHNW: metabolically healthy normal weight; MHO: metabolically healthy obese; MHOW: metabolically healthy overweight; MUNW: metabolically unhealthy normal weight; MUO: metabolically unhealthy obese; MUOW: metabolically unhealthy overweight.

Subgroup analyses were conducted by providing further evidence of a sex factor in the BN model; these analyses are summarized in Figure 4. In men, the conditional probabilities of high CVD risk were nearly doubled among participants who were MH, regardless of BMI level, with probabilities ranging from 12.28% to 21.92% for men who were MHNW and MHO, respectively. This indicates that men who were MH had twice the chance of high CVD risk when compared with their general population counterparts within the same obesity phenotype. Similarly, the men who were MU also had an increased CVD risk, where male participants who were MUO were nearly 2-fold more likely to have a high CVD risk than their MHO counterparts, with a conditional probability up to 48.21% (95% CI 42.92 to 53.55), whereas these probabilities were raised by a factor of ≥2.5 among men who were MUNW and MUOW when compared with their MH counterparts within the same BMI levels (MUNW vs MHNW: 32.18% vs 12.28%; MUOW vs MHOW: 43.17% vs 15.4%).

In contrast, female participants with favorable metabolic health profiles had substantially lower conditional probability estimates of moderate and high CVD risk, irrespective of their BMI level, with probabilities of only 10.61% and 2.02% in women who were MHO. Similarly, only approximately one-fifth of the women who were MUO (22.7%, 95% CI 18.83-27.11) and one-tenth of the women who were MU and nonobese (13% for women who were MUNW and 11.91% for women who were MUOW) had a high risk of developing CVD, whereas women who were MH (irrespective of obesity) were more than half as likely to have a low CVD risk, with corresponding conditional probabilities of 64.74% (95% CI 61.43 to 67.92), 58.84% (95% CI 55.5 to 62.11), and 54.08% (95% CI 49.13-58-95) for women who were MUNW, MUOW, and MUO, respectively.

## Discussion

### Principal Findings

To the best of our knowledge, this is the first large-scale study that has applied the BN modeling approach to investigate the probabilistic relationship between different metabolic and obesity phenotypes and the 10-year CVD risk in Chinese adults. Individuals who were MHO had an increased probability (10.47%) of high CVD risk, and this probability doubled among participants who were MUNW and tripled for participants who were MUO. Furthermore, an important gap in conditional probabilities was found between the two sexes within each obesity phenotype, suggesting a prominent modifying effect of sex on this relationship. The proposed DAG structure in the network represents a relevant step in understanding the complex interrelationships between the variables investigated and provides a self-descriptive and contextualized picture of these complex interrelationships in the Chinese population.

Compared with previous studies, this work offers a more comprehensive picture that simultaneously describes the complex interrelationships between metabolic healthy or unhealthy phenotypes and 10-year CVD risk across BMI categories, as well as the relationships among CVD-related risk factors. Although several prospective cohort studies have focused on exploring the specific associations between CVD risk and metabolic health status and BMI categories [14,45-47], or the dynamic risk in the transition from one phenotype to another [48-50], this work aimed to provide a general framework to understand the multiple association processes that can emerge from the complex interrelationships between these factors. In fact, compared with standard studies, the greatest advantage and strength of this BN methodological approach is the graphical clarification and visualization of the most probable pathways in these relationships from a multi-dependent perspective, without affecting the interpretability of the network. Another advantage of BN modeling is that owing to the Markov blanket theory, complex models can be divided into a collection of simpler models that are mathematically tractable and computationally simpler. For instance, according to our BN reasoning model (Figure 1B), when a participant who was MH and normal weight became obese, the probability of developing a high 10-year CVD level increased from 7.01% to 10.47%, and this probability was tripled if the participant remained normal weight but had any ≥2 components of MetS. Furthermore, the probability increases up to 34.48% when the participant has both a MU status and is obese (ie, MUO; Figure 2). Moreover, remarkable heterogeneity in the associations between obesity phenotypes and CVD risk in relation to participant sex was observed. The conditional probability of having a high CVD risk was 2.02% and 22.7% among women who were MHO and MUO, respectively, whereas it rose to as high as 21.92% and 48.21% among men with the corresponding obesity phenotypes. Therefore, BN modeling enabled us to achieve an integrated view of CVD risk among the various obesity phenotypes in the context of other risk factors. It also allows for systematic reasoning in the diagnostic process with easy interpretability.

### Comparison With Prior Work

Many studies have investigated the associations between obesity phenotypes and CVD outcomes, including myocardial infarction [51], HF [45,52], coronary heart disease [45,53], atrial fibrillation [54], and cerebrovascular disease [45]. This study confirms an elevated risk of CVD in people classified as MHOW or MHO when compared with their counterparts who are of normal weight and are MH. This is in line with previous studies in Asian [50,55], European [46], and American populations [56]. In the Danish prospective Inter99 study, Hansen et al [47] found that men who were MHO had a 3-fold increased risk of incident CVD compared with their MHNW counterparts, and a similar and significant augmentation of CVD risk was also observed in men who were MU, with a 2.2-fold increased risk in men who were MUNW and an approximately 3-fold increase in men who were MUOW and MUO, respectively, during the 10-year follow-up. In contrast, the increased risk among women who were MH was not significant, irrespective of BMI level, when compared with their MHNW counterparts. In fact, an inverted U-shaped relationship was observed between women who were MU and CVD risk across the BMI levels, and a significant 2.3-fold increased risk was only observed among women who were MUOW compared with women who were MHNW [47]. The Nurses’ Health Study, which included 90,257 American women aged 30 to 55 years without CVD or cancer history [56], indicated that women with either higher BMI levels or MU status were at a significantly increased risk compared with their MHNW counterparts after a follow-up of 30 years. In addition, women who were MH had a substantially lower risk of CVD than women with pre-existing metabolic conditions across all BMI groups. Consistently, the Whitehall 2 study also found that, compared with the MHNW phenotype, the risk of incident CVD was significantly elevated in the 5 other phenotypes during a median follow-up of 17.4 years, with adjusted hazard ratios (HRs) of 1.95 for MHO and 2.44 for MUO. Similarly, the participants who were MU had a higher risk than their MH counterparts, irrespective of BMI levels, by a factor of 2 for the nonobese BMI level and 1.2 for the obese BMI level [46]. Similarly, the findings from several Chinese prospective cohort studies, with either short- or long-term follow-ups [48,57,58], are consistent with those obtained in Western populations. The largest prospective cohort study, the China Kadoorie Biobank study, which comprised 458,246 Chinese participants without any history of CVD or cancer, found that after 10 years of follow-up, individuals who were baseline MHO had an 8% higher risk of developing CVD compared with their MHNW counterparts, and the risk for individuals who were MU was significantly higher across all BMI categories by a factor of 1.6 [48]. Of note, a very recent meta-analysis of 23 prospective cohort studies and 4,492,723 participants confirmed an elevated risk of CVD in individuals classified as MHOW or MHO when compared with their MHNW counterparts by a factor of 1.34 and 1.5, respectively. This increased risk remained statistically significant among individuals who were MHOW or MHO when defining metabolic health status with a strict definition (ie, having no metabolic risk factors) [14]. This indicates the potential nonexistence of the MHOW or MHO concept. Another important observation, as described in the meta-analyses by Kramer et al [59], Fan et al [60], Eckel et al [61], Zheng et al [62], Ortega et al [63], and others, is that the high risk of CVD associated with MHO appears to be sustained over a long-term follow-up (≥15 years). Several mechanisms could explain the potential association between MHO and the risk of CVD. Individuals who were MHO or MHOW may have higher odds of subclinical CVD and diabetes, which increases their likelihood of developing CVD in the future [64].

Smoking status and age have well-known causal effects on long-term CVD risk [65-67]. The current results suggest that smoking status and age are directly associated with 10-year CVD risk and seem to mediate the effect of sex and other variables included in our BN model. To the best of our knowledge, few studies have focused on sex-specific differences in the relationship between obesity phenotypes and CVD risk, although sex is a very important factor [68,69]. In this, men who were obese had an elevated probability of high 10-year CVD risk when compared with their female peers, irrespective of metabolic health status. Although there are some discrepancies in the way that obesity and metabolic health status have been defined, the current findings of sex-specific differences are consistent with other large prospective studies in Chinese, European, and US samples [47,48,70]. For instance, Danish men who were MHO and MUO had a 3.1- and 2.7-fold increased CVD risk compared with their MHNW counterparts, whereas this increased risk was only by a factor of 1.8 in women within the same comparison of phenotypes [47]. Similarly, the China Kadoorie Biobank study found that men had a 1.09 times higher risk of CVD subtypes when compared with women within the MHO phenotype and a 1.3 times higher increased risk within the MUO phenotype [48]. This finding was also supported by a recent pooled analysis of prospective cohort studies, which revealed that men who were MHO had a 1.26 times increased risk compared with women who were MHO (HR: 2.15 vs 1.71) [14].

The present results are also in agreement with those of previous cohort studies that described the progression of CVD risk with aging. The China Kadoorie Biobank study demonstrated a clear age-specific pattern in CVD risk, irrespective of obesity phenotypes [48]. A steady rise in CVD risk was noted from age 30 to 49 years to 50 to 59 years, and this risk was substantially increased for individuals aged ≥60 years within each obesity phenotype. Individuals who were MUO aged 70 to 79 years had the highest risk of developing CVD events among all obesity phenotypes, with a 13.86-fold higher risk when taking individuals with MHNW at age 30 to 49 years as the reference group. Similarly, this risk was higher among participants who were MHNW aged ≥70 years compared with their counterparts aged 30 to 49 years. The current BN model applied to a population-based Chinese cohort showed a concave-shaped progression in the conditional probabilities for high CVD risk through the different age intervals together with a stronger increasing rate in the conditional probability after the age of 50 years (Figure 2). Clearly, the use of such BN modeling with respect to prior knowledge could provide quantitative descriptions of direct links between CVD and its related risk factors by intuitive reasoning. More importantly, such modeling is suited to exploring indirect links through mediators and testing novel hypotheses by simulation.

The CVD risk in individuals who are MU with normal weight remains underinvestigated. A large pan-European prospective study of 8 European countries found that after a median follow-up of 12.2 years [53], the presence of metabolic abnormalities was associated with an increased risk of CHD at all levels of adiposity; more precisely, the MUNW phenotype had twice the risk of CHD compared with their MH counterparts. This finding is supported by recent data from the Women’s Health Initiative Study [70] and a Korean prospective study [54]. Interestingly, several studies have demonstrated that the CVD risk in individuals who are MU is markedly higher than that of their MH counterparts across all BMI categories [53]. Similarly, when using the MHO group as a reference, the MU nonobese group was found to be at increased risk of atrial fibrillation, although this difference was not statistically significant [54]. The current results are in agreement with previous studies and contribute to the evidence indicating that individuals who are MUNW are at considerably higher CVD risk compared with their peers who are MHO, regardless of sex, and it seems reasonable to suggest that individuals who are overweight or obese without metabolic abnormalities are at intermediate CVD risk, at a level between individuals who are healthy normal weight and individuals who are MU [53].

Another potential explanation for these findings may be related to the transient status of MHO during long-term follow-up. Indeed, several recent studies have considered and identified CVD risk according to the concurrent transition of metabolic health and weight status during different follow-up periods [49,50,56]. In one study, the recovery of MH status among individuals who were baseline MUNO was significantly associated with a decreased risk of CVD outcomes, whereas the transition from an MH status to an unhealthy status among the individuals who were baseline MUNO was related to adverse CVD outcomes [50]. These findings were also confirmed by Bae et al [49] using a nationally representative cohort study of 205,394 middle-aged Korean men and women who were followed up for 6 years. Interestingly, among the initial participants who were MHO, those who became MUNO had a 1.41-fold higher CVD risk compared with those who remained MHO. Together, this evidence strongly suggests a greater role of MetS than obesity in CVD risk, with longer exposure to a MU status leading to a much higher vascular risk [48]. Moreover, another nationwide population-based cohort study revealed that transition to the MUNO phenotype was associated with an 80% higher HR for HF among individuals who were MHO at baseline during a short-term follow-up (3.7 years). Conversely, individuals who transitioned from MHO to MH nonobese had a lower HR for HF than those who remained in the MHO category [52]. This could imply that restoration from an obese state to a nonobese state while maintaining metabolic health may have a protective effect against incident HF. However, as emphasized by Gao et al [48] in the largest Asian cohort study to date on the transitions between various obesity phenotypes over a longer follow-up, long-term maintenance of metabolic health is difficult for individuals of any BMI level, including individuals who are overweight and normal weight; therefore, more attention should be paid to maintaining metabolic health regardless of body weight. In addition, there should be a clinical focus on the treatment of metabolic disorders for CVD risk prevention. Similarly, efforts should be made to prevent the conversion of MHO to MUO and the development of MetS and subsequent CVD caused by obesity [71,72].

### Limitations

There are several limitations to this study and the newly identified networks that should be noted. First, despite the longitudinal design of the CHNS survey, this study analyzed observational and cross-sectional data; the directions between nodes or variables only represent probability dependencies, not causal relationships. Further cohort studies combined with various aspects of professional knowledge are warranted to establish and clarify causality [73]. In addition, the sample only comprised Chinese participants; thus, the generalizability of these results to a wider population should be undertaken with caution. In addition, physical activity and fitness were lacking in the proposed BN modeling, although these 2 factors may be more important than weight in assessing CVD risk, as emphasized by Lavie et al [74]. In fact, the information on physical activity and fitness was not exhaustively provided from the CHNS data; thus, they were not included in this study for minimizing the potential bias but will be well-considered in further studies.

Nevertheless, the notable strengths and contributions of this study should be mentioned. For example, the population-based design, large sample size, and rigorous data collection quality guarantee reasonable statistical power and robust probabilistic relationships. Second, the BN modeling approach offers compelling application prospects in general medicine. BN is not only useful for handling a large number of variables with or without prior knowledge of the interactions or interdependencies between them [38] but also provides an appealing visual presentation and quantitative reasoning that can be used to explore the interrelationships among these factors and test novel hypotheses.

### Conclusions

The BN modeling approach was applied to investigate the relationships between different CVD risk factors and metabolic health and obesity status using Chinese population-based survey data. Network modeling is useful for integrating expert knowledge and observational data, allowing easy identification of probabilistic dependencies and conditional independencies between variables through graphical representation. This study provides evidence that increased CVD risk progresses depending on the varying magnitude of metabolic abnormalities and BMI. Furthermore, several potential modifying factors were identified, including sex, that may affect previous probabilistic interrelationships. Owing to the multifactorial nature of CVD, these empirical findings using the BN approach are of special interest, both from a theoretical and practical point of view, and may help in refining appropriate target populations and relevant risk factors for managing future CVD risk.

## Appendix

Multimedia Appendix 1 The directed acyclic (DAG) graph underlying the Bayesian network learned from 10-year cardiovascular disease risk, covariates, metabolic health, and obesity status. (A) Averaged DAG with arcs >0.5; (B) simplified DAG derived from the averaged DAG after retaining arcs with a strength >0.85.

## Abbreviations

BN: Bayesian network
BP: blood pressure
CHNS: China Health and Nutrition Survey
CVD: cardiovascular disease
DAG: directed acyclic graph
DBP: diastolic blood pressure
eGFR: estimated glomerular filtration rate
FPG: fasting plasma glucose
FRS: Framingham Risk Score
HDL-C: high-density lipoprotein cholesterol
HF: heart failure
HOMA-IR: homeostasis model assessment of insulin resistance
HR: hazard ratio
MetS: metabolic syndrome
MH: metabolically healthy
MHNW: metabolically healthy normal weight
MHO: metabolically healthy obese
MHOW: metabolically healthy overweight
MU: metabolically unhealthy
MUNW: metabolically unhealthy normal weight
MUO: metabolically unhealthy obese
MUOW: metabolically unhealthy overweight
SBP: systolic blood pressure
TC: total cholesterol
TG: triglyceride
UA: uric acid

## References

[1] Roth GA, Mensah GA, Johnson CO, Addolorato G, Ammirati E, Baddour LM, Barengo NC, Beaton AZ, Benjamin EJ, Benziger CP, Bonny A, Brauer M, Brodmann M, Cahill TJ, Carapetis J, Catapano AL, Chugh SS, Cooper LT, Coresh J, Criqui M, DeCleene N, Eagle KA, Emmons-Bell S, Feigin VL, Fernández-Solà J, Fowkes G, Gakidou E, Grundy SM, He FJ, Howard G, Hu F, Inker L, Karthikeyan G, Kassebaum N, Koroshetz W, Lavie C, Lloyd-Jones D, Lu HS, Mirijello A, Temesgen AM, Mokdad A, Moran AE, Muntner P, Narula J, Neal B, Ntsekhe M, Moraes de Oliveira G, Otto C, Owolabi M, Pratt M, Rajagopalan S, Reitsma M, Ribeiro AL, Rigotti N, Rodgers A, Sable C, Shakil S, Sliwa-Hahnle K, Stark B, Sundström J, Timpel P, Tleyjeh IM, Valgimigli M, Vos T, Whelton PK, Yacoub M, Zuhlke L, Murray C, Fuster V, GBD-NHLBI-JACC Global Burden of Cardiovascular Diseases Writing Group (2020). Global burden of cardiovascular diseases and risk factors, 1990-2019: update from the GBD 2019 study. J Am Coll Cardiol.

[2] Zhao D, Liu J, Wang M, Zhang X, Zhou M (2019). Epidemiology of cardiovascular disease in China: current features and implications. Nat Rev Cardiol.

[3] Liu S, Li Y, Zeng X, Wang H, Yin P, Wang L, Liu Y, Liu J, Qi J, Ran S, Yang S, Zhou M (2019). Burden of cardiovascular diseases in China, 1990-2016: findings from the 2016 global burden of disease study. JAMA Cardiol.

[4] Yatsuya H, Li Y, Hilawe EH, Ota A, Wang C, Chiang C, Zhang Y, Uemura M, Osako A, Ozaki Y, Aoyama A (2014). Global trend in overweight and obesity and its association with cardiovascular disease incidence. Circ J.

[5] O'Brien EC, Fosbol EL, Peng SA, Alexander KP, Roe MT, Peterson ED (2014). Association of body mass index and long-term outcomes in older patients with non-ST-segment-elevation myocardial infarction: results from the CRUSADE Registry. Circ Cardiovasc Qual Outcomes.

[6] Lahey R, Khan SS (2018). Trends in obesity and risk of cardiovascular disease. Curr Epidemiol Rep.

[7] Chen Z, Iona A, Parish S, Chen Y, Guo Y, Bragg F, Yang L, Bian Z, Holmes MV, Lewington S, Lacey B, Gao R, Liu F, Zhang Z, Chen J, Walters RG, Collins R, Clarke R, Peto R, Li L, China Kadoorie Biobank collaborative group (2018). Adiposity and risk of ischaemic and haemorrhagic stroke in 0·5 million Chinese men and women: a prospective cohort study. Lancet Glob Health.

[8] Alberti KG, Eckel RH, Grundy SM, Zimmet PZ, Cleeman JI, Donato KA, Fruchart J, James WP, Loria CM, Smith SC, International Diabetes Federation Task Force on EpidemiologyPrevention, Hational Heart‚ Lung‚Blood Institute, American Heart Association, World Heart Federation, International Atherosclerosis Society, International Association for the Study of Obesity (2009). Harmonizing the metabolic syndrome: a joint interim statement of the International Diabetes Federation Task Force on Epidemiology and Prevention; National Heart, Lung, and Blood Institute; American Heart Association; World Heart Federation; International Atherosclerosis Society; and International Association for the Study of Obesity. Circulation.

[9] Stefan N, Häring H-U, Hu FB, Schulze MB (2013). Metabolically healthy obesity: epidemiology, mechanisms, and clinical implications. Lancet Diabetes Endocrinol.

[10] Yang HK, Han K, Kwon H, Park Y, Cho J, Yoon K, Kang M, Cha B, Lee S (2016). Obesity, metabolic health, and mortality in adults: a nationwide population-based study in Korea. Sci Rep.

[11] Montalescot G, Sechtem U, Achenbach S, Andreotti F, Arden C, Budaj A, Bugiardini R, Crea F, Cuisset T, Di Mario C, Ferreira JR, Gersh BJ, Gitt AK, Hulot J, Marx N, Opie LH, Pfisterer M, Prescott E, Ruschitzka F, Sabaté M, Senior R, Taggart DP, van der Wall EE, Vrints CJ, Zamorano JL, Achenbach S, Baumgartner H, Bax JJ, Bueno H, Dean V, Deaton C, Erol C, Fagard R, Ferrari R, Hasdai D, Hoes AW, Kirchhof P, Knuuti J, Kolh P, Lancellotti P, Linhart A, Nihoyannopoulos P, Piepoli MF, Ponikowski P, Sirnes PA, Tamargo JL, Tendera M, Torbicki A, Wijns W, Windecker S, Knuuti J, Valgimigli M, Bueno H, Claeys MJ, Donner-Banzhoff N, Erol C, Frank H, Funck-Brentano C, Gaemperli O, Gonzalez-Juanatey JR, Hamilos M, Hasdai D, Husted S, James SK, Kervinen K, Kolh P, Kristensen SD, Lancellotti P, Maggioni AP, Piepoli MF, Pries AR, Romeo F, Rydén L, Simoons ML, Sirnes PA, Steg PG, Timmis A, Wijns W, Windecker S, Yildirir A, Zamorano JL, Task Force Members, ESC Committee for Practice Guidelines, Document Reviewers (2013). 2013 ESC guidelines on the management of stable coronary artery disease: the Task Force on the management of stable coronary artery disease of the European Society of Cardiology. Eur Heart J.

[12] Mozaffarian D, Benjamin EJ, Go AS, Arnett DK, Blaha MJ, Cushman M, Das SR, de Ferranti S, Després J-P, Fullerton HJ, Howard VJ, Huffman MD, Isasi CR, Jiménez MC, Judd SE, Kissela BM, Lichtman JH, Lisabeth LD, Liu S, Mackey RH, Magid DJ, McGuire DK, Mohler ER, Moy CS, Muntner P, Mussolino ME, Nasir K, Neumar RW, Nichol G, Palaniappan L, Pandey DK, Reeves MJ, Rodriguez CJ, Rosamond W, Sorlie PD, Stein J, Towfighi A, Turan TN, Virani SS, Woo D, Yeh RW, Turner MB, Writing Group Members, American Heart Association Statistics Committee, Stroke Statistics Subcommittee (2016). Executive summary: heart disease and stroke statistics--2016 update: a report from the American Heart Association. Circulation.

[13] Badawi A, Di Giuseppe G, Arora P (2018). Cardiovascular disease risk in patients with hepatitis C infection: results from two general population health surveys in Canada and the United States (2007-2017). PLoS One.

[14] Opio J, Croker E, Odongo GS, Attia J, Wynne K, McEvoy M (2020). Metabolically healthy overweight/obesity are associated with increased risk of cardiovascular disease in adults, even in the absence of metabolic risk factors: a systematic review and meta-analysis of prospective cohort studies. Obes Rev.

[15] Scutari M, Vitolo C, Tucker A (2019). Learning Bayesian networks from big data with greedy search: computational complexity and efficient implementation. Stat Comput.

[16] Jensen F, Nielsen T (2001). Bayesian Networks and Decision Graphs.

[17] Gupta A, Slater JJ, Boyne D, Mitsakakis N, Béliveau A, Druzdzel MJ, Brenner DR, Hussain S, Arora P (2019). Probabilistic graphical modeling for estimating risk of coronary artery disease: applications of a flexible machine-learning method. Med Decis Making.

[18] Fuster-Parra P, Tauler P, Bennasar-Veny M, Ligęza A, López-González AA, Aguiló A (2016). Bayesian network modeling: a case study of an epidemiologic system analysis of cardiovascular risk. Comput Methods Programs Biomed.

[19] Badawi A, Di Giuseppe G, Gupta A, Poirier A, Arora P (2020). Bayesian network modelling study to identify factors influencing the risk of cardiovascular disease in Canadian adults with hepatitis C virus infection. BMJ Open.

[20] Yan S, Li J, Li S, Zhang B, Du S, Gordon-Larsen P, Adair L, Popkin B (2012). The expanding burden of cardiometabolic risk in China: the China Health and Nutrition Survey. Obes Rev.

[21] Tian S, Liu Y, Feng A, Zhang S (2020). Sex-specific differences in the association of metabolically healthy obesity with hyperuricemia and a network perspective in analyzing factors related to hyperuricemia. Front Endocrinol (Lausanne).

[22] Ye X, Liu X, Song D, Zhang X, Zhu B, Wei L, Pei X, Wu J, Lou T, Zhao W (2016). Estimating glomerular filtration rate by serum creatinine or/and cystatin C equations: an analysis of multi-centre Chinese subjects. Nephrology (Carlton).

[23] James PA, Oparil S, Carter BL, Cushman WC, Dennison-Himmelfarb C, Handler J, Lackland DT, LeFevre ML, MacKenzie TD, Ogedegbe O, Smith SC, Svetkey LP, Taler SJ, Townsend RR, Wright JT, Narva AS, Ortiz E (2014). 2014 evidence-based guideline for the management of high blood pressure in adults: report from the panel members appointed to the Eighth Joint National Committee (JNC 8). JAMA.

[24] Liu L, Writing Group of 2010 Chinese Guidelines for the Management of Hypertension (2011). [2010 Chinese guidelines for the management of hypertension]. Zhonghua Xin Xue Guan Bing Za Zhi.

[25] National Cholesterol Education Program (NCEP) Expert Panel on Detection‚ Evaluation‚Treatment of High Blood Cholesterol in Adults (Adult Treatment Panel III) (2002). Third report of the national cholesterol education program (NCEP) expert panel on detection, evaluation, and treatment of high blood cholesterol in adults (adult treatment panel III) final report. Circulation.

[26] Zhang W, Doherty M, Bardin T, Pascual E, Barskova V, Conaghan P, Gerster J, Jacobs J, Leeb B, Lioté F, McCarthy G, Netter P, Nuki G, Perez-Ruiz F, Pignone A, Pimentão J, Punzi L, Roddy E, Uhlig T, Zimmermann-Gòrska I, EULAR Standing Committee for International Clinical Studies Including Therapeutics (2006). EULAR evidence based recommendations for gout. Part II: management. Report of a task force of the EULAR Standing Committee for International Clinical Studies Including Therapeutics (ESCISIT). Ann Rheum Dis.

[27] D'Agostino RB, Vasan RS, Pencina MJ, Wolf PA, Cobain M, Massaro JM, Kannel WB (2008). General cardiovascular risk profile for use in primary care: the Framingham Heart Study. Circulation.

[28] Bosomworth NJ (2011). Practical use of the Framingham risk score in primary prevention: Canadian perspective. Can Fam Physician.

[29] Anderson TJ, Grégoire J, Pearson GJ, Barry AR, Couture P, Dawes M, Francis GA, Genest J, Grover S, Gupta M, Hegele RA, Lau DC, Leiter LA, Lonn E, Mancini GJ, McPherson R, Ngui D, Poirier P, Sievenpiper JL, Stone JA, Thanassoulis G, Ward R (2016). 2016 canadian cardiovascular society guidelines for the management of dyslipidemia for the prevention of cardiovascular disease in the adult. Can J Cardiol.

[30] Novo S, Carità P, Lo Voi A, Muratori I, Tantillo R, Corrado E, Kalodiki E, Novo G (2019). Impact of preclinical carotid atherosclerosis on global cardiovascular risk stratification and events in a 10-year follow-up: comparison between the algorithms of the Framingham Heart Study, the European SCORE and the Italian 'Progetto Cuore'. J Cardiovasc Med (Hagerstown).

[31] Jamthikar A, Gupta D, Cuadrado-Godia E, Puvvula A, Khanna NN, Saba L, Viskovic K, Mavrogeni S, Turk M, Laird JR, Pareek G, Miner M, Sfikakis PP, Protogerou A, Kitas GD, Shankar C, Nicolaides A, Viswanathan V, Sharma A, Suri JS (2020). Ultrasound-based stroke/cardiovascular risk stratification using Framingham Risk Score and ASCVD Risk Score based on "Integrated Vascular Age" instead of "Chronological Age": a multi-ethnic study of Asian Indian, Caucasian, and Japanese cohorts. Cardiovasc Diagn Ther.

[32] Zhou J, Gao Q, Wang J, Zhang M, Ma J, Wang C, Chen H, Peng X, Hao L (2018). Comparison of coronary heart disease risk assessments among individuals with metabolic syndrome using three diagnostic definitions: a cross-sectional study from China. BMJ Open.

[33] Shillinglaw B, Viera AJ, Edwards T, Simpson R, Sheridan SL (2012). Use of global coronary heart disease risk assessment in practice: a cross-sectional survey of a sample of U.S. physicians. BMC Health Serv Res.

[34] Chen C, Lu FC, Department of Disease Control Ministry of Health‚ PR China (2004). The guidelines for prevention and control of overweight and obesity in Chinese adults. Biomed Environ Sci.

[35] (2000). The Asia-pacific Perspective : Redefining Obesity and Its Treatment.

[36] Expert Panel on Detection‚ Evaluation‚Treatment of High Blood Cholesterol in Adults (2001). Executive Summary of The Third Report of The National Cholesterol Education Program (NCEP) expert panel on detection, evaluation, and treatment of high blood cholesterol in adults (Adult Treatment Panel III). JAMA.

[37] Constantinou AC, Fenton N, Marsh W, Radlinski L (2016). From complex questionnaire and interviewing data to intelligent Bayesian network models for medical decision support. Artif Intell Med.

[38] Chao Y, Wu H, Scutari M, Chen T, Wu C, Durand M, Boivin A (2017). A network perspective on patient experiences and health status: the Medical Expenditure Panel Survey 2004 to 2011. BMC Health Serv Res.

[39] Amirkhani H, Rahmati M, Lucas PJ, Hommersom A (2017). Exploiting experts' knowledge for structure learning of Bayesian networks. IEEE Trans Pattern Anal Mach Intell.

[40] Sambo F, Di Camillo B, Franzin A, Facchinetti A, Hakaste L, Kravic J, Fico G, Tuomilehto J, Groop L, Gabriel R, Tuomi T, Cobelli C (2015). A Bayesian Network analysis of the probabilistic relations between risk factors in the predisposition to type 2 diabetes. Annu Int Conf IEEE Eng Med Biol Soc.

[41] Glover F (1990). Artificial intelligence, heuristic frameworks and tabu search. Manage Decision Econ.

[42] Schwarz G (1978). Estimating the dimension of a model. Ann Stat.

[43] Scutari M (2010). Learning Bayesian networks with the package. J Stat Softw.

[44] (2020). R: A Language and Environment for Statistical Computing.

[45] Caleyachetty R, Thomas GN, Toulis KA, Mohammed N, Gokhale KM, Balachandran K, Nirantharakumar K (2017). Metabolically healthy obese and incident cardiovascular disease events among 3.5 million men and women. J Am Coll Cardiol.

[46] Hinnouho G, Czernichow S, Dugravot A, Nabi H, Brunner EJ, Kivimaki M, Singh-Manoux A (2015). Metabolically healthy obesity and the risk of cardiovascular disease and type 2 diabetes: the Whitehall II cohort study. Eur Heart J.

[47] Hansen L, Netterstrøm MK, Johansen NB, Rønn PF, Vistisen D, Husemoen LL, Jørgensen ME, Rod NH, Færch K (2017). Metabolically healthy obesity and ischemic heart disease: a 10-year follow-up of the Inter99 study. J Clin Endocrinol Metab.

[48] Gao M, Lv J, Yu C, Guo Y, Bian Z, Yang R, Du H, Yang L, Chen Y, Li Z, Zhang X, Chen J, Qi L, Chen Z, Huang T, Li L, China Kadoorie Biobank (CKB) Collaborative Group (2020). Metabolically healthy obesity, transition to unhealthy metabolic status, and vascular disease in Chinese adults: a cohort study. PLoS Med.

[49] Bae YS, Choi S, Lee K, Son JS, Lee H, Cho MH, Koo H, Cho IY, Chang J, Kim K, Kim SM, Park SM (2019). Association of concurrent changes in metabolic health and weight on cardiovascular disease risk: a nationally representative cohort study. J Am Heart Assoc.

[50] Cho YK, Kang YM, Yoo JH, Lee J, Park J, Lee WJ, Kim Y, Jung CH (2019). Implications of the dynamic nature of metabolic health status and obesity on risk of incident cardiovascular events and mortality: a nationwide population-based cohort study. Metabolism.

[51] Mirzababaei A, Djafarian K, Mozafari H, Shab-Bidar S (2019). The long-term prognosis of heart diseases for different metabolic phenotypes: a systematic review and meta-analysis of prospective cohort studies. Endocrine.

[52] Lee Y, Kim DH, Kim SM, Kim NH, Choi KM, Baik SH, Park YG, Han K, Yoo HJ (2020). Hospitalization for heart failure incidence according to the transition in metabolic health and obesity status: a nationwide population-based study. Cardiovasc Diabetol.

[53] Lassale C, Tzoulaki I, Moons KG, Sweeting M, Boer J, Johnson L, Huerta JM, Agnoli C, Freisling H, Weiderpass E, Wennberg P, van der AL, Arriola L, Benetou V, Boeing H, Bonnet F, Colorado-Yohar SM, Engström G, Eriksen AK, Ferrari P, Grioni S, Johansson M, Kaaks R, Katsoulis M, Katzke V, Key TJ, Matullo G, Melander O, Molina-Portillo E, Moreno-Iribas C, Norberg M, Overvad K, Panico S, Quirós JR, Saieva C, Skeie G, Steffen A, Stepien M, Tjønneland A, Trichopoulou A, Tumino R, van der Schouw YT, Verschuren WM, Langenberg C, Di Angelantonio E, Riboli E, Wareham NJ, Danesh J, Butterworth AS (2018). Separate and combined associations of obesity and metabolic health with coronary heart disease: a pan-European case-cohort analysis. Eur Heart J.

[54] Lee H, Choi E, Lee S, Han K, Rhee T, Park C, Lee S, Choe W, Lim W, Kang S, Cha M, Oh S (2017). Atrial fibrillation risk in metabolically healthy obesity: a nationwide population-based study. Int J Cardiol.

[55] Huang M, Wang M, Lin Y, Lin C, Lo K, Chang I, Cheng T, Tsai S, Chen H, Lin C, Liu SJ, Chien K, Yeh T (2020). The association between metabolically healthy obesity, cardiovascular disease, and all-cause mortality risk in Asia: a systematic review and meta-analysis. Int J Environ Res Public Health.

[56] Eckel N, Li Y, Kuxhaus O, Stefan N, Hu FB, Schulze MB (2018). Transition from metabolic healthy to unhealthy phenotypes and association with cardiovascular disease risk across BMI categories in 90 257 women (the Nurses' Health Study): 30 year follow-up from a prospective cohort study. Lancet Diabetes Endocrinol.

[57] Li L, Chen K, Wang A, Gao J, Zhao K, Wang H, Dou J, Lv Z, Wang B, Yan W, Yang L, Mu Y (2018). Cardiovascular disease outcomes in metabolically healthy obesity in communities of Beijing cohort study. Int J Clin Pract.

[58] Li H, He D, Zheng D, Amsalu E, Wang A, Tao L, Guo J, Li X, Wang W, Guo X (2019). Metabolically healthy obese phenotype and risk of cardiovascular disease: results from the China Health and Retirement Longitudinal Study. Arch Gerontol Geriatr.

[59] Kramer CK, Zinman B, Retnakaran R (2013). Are metabolically healthy overweight and obesity benign conditions?: a systematic review and meta-analysis. Ann Intern Med.

[60] Fan J, Song Y, Chen Y, Hui R, Zhang W (2013). Combined effect of obesity and cardio-metabolic abnormality on the risk of cardiovascular disease: a meta-analysis of prospective cohort studies. Int J Cardiol.

[61] Eckel N, Meidtner K, Kalle-Uhlmann T, Stefan N, Schulze MB (2016). Metabolically healthy obesity and cardiovascular events: a systematic review and meta-analysis. Eur J Prev Cardiol.

[62] Zheng R, Zhou D, Zhu Y (2016). The long-term prognosis of cardiovascular disease and all-cause mortality for metabolically healthy obesity: a systematic review and meta-analysis. J Epidemiol Community Health.

[63] Ortega FB, Cadenas-Sanchez C, Migueles JH, Labayen I, Ruiz JR, Sui X, Blair SN, Martínez-Vizcaino V, Lavie CJ (2018). Role of physical activity and fitness in the characterization and prognosis of the metabolically healthy obesity phenotype: a systematic review and meta-analysis. Prog Cardiovasc Dis.

[64] Dwivedi AK, Dubey P, Cistola DP, Reddy SY (2020). Association between obesity and cardiovascular outcomes: updated evidence from meta-analysis studies. Curr Cardiol Rep.

[65] Banks E, Joshy G, Korda RJ, Stavreski B, Soga K, Egger S, Day C, Clarke NE, Lewington S, Lopez AD (2019). Tobacco smoking and risk of 36 cardiovascular disease subtypes: fatal and non-fatal outcomes in a large prospective Australian study. BMC Med.

[66] Duncan MS, Freiberg MS, Greevy RA, Kundu S, Vasan RS, Tindle HA (2019). Association of smoking cessation with subsequent risk of cardiovascular disease. JAMA.

[67] Costantino S, Paneni F, Cosentino F (2016). Ageing, metabolism and cardiovascular disease. J Physiol.

[68] Ventura-Clapier R, Dworatzek E, Seeland U, Kararigas G, Arnal J, Brunelleschi S, Carpenter TC, Erdmann J, Franconi F, Giannetta E, Glezerman M, Hofmann SM, Junien C, Katai M, Kublickiene K, König IR, Majdic G, Malorni W, Mieth C, Miller VM, Reynolds RM, Shimokawa H, Tannenbaum C, D'Ursi AM, Regitz-Zagrosek V (2017). Sex in basic research: concepts in the cardiovascular field. Cardiovasc Res.

[69] Appelman Y, van Rijn BB, Ten Haaf ME, Boersma E, Peters SA (2015). Sex differences in cardiovascular risk factors and disease prevention. Atherosclerosis.

[70] Chen G, Arthur R, Iyengar NM, Kamensky V, Xue X, Wassertheil-Smoller S, Allison MA, Shadyab AH, Wild RA, Sun Y, Banack HR, Chai JC, Wactawski-Wende J, Manson JE, Stefanick ML, Dannenberg AJ, Rohan TE, Qi Q (2019). Association between regional body fat and cardiovascular disease risk among postmenopausal women with normal body mass index. Eur Heart J.

[71] Lavie CJ, Deedwania P, Ortega FB (2018). Obesity is rarely healthy. Lancet Diabetes Endocrinol.

[72] Deedwania P, Lavie CJ (2018). Dangers and long-term outcomes in metabolically healthy obesity: the impact of the missing fitness component. J Am Coll Cardiol.

[73] Nagarajan R, Scutari M, Lèbre S (2013). Bayesian Networks in R.

[74] Lavie CJ, Ozemek C, Carbone S, Katzmarzyk PT, Blair SN (2019). Sedentary behavior, exercise, and cardiovascular health. Circ Res.

