# Supplementary figures and images for: A Bayesian Network Analysis of the Probabilistic Relationships Between Various Obesity Phenotypes and Cardiovascular Disease Risk in Chinese Adults: Chinese Population-Based Observational Study
Source: JMIR Med Inform. 2022 Mar 2;10(3):e33026. doi: 10.2196/33026 (PMC8928047; doi:10.2196/33026)

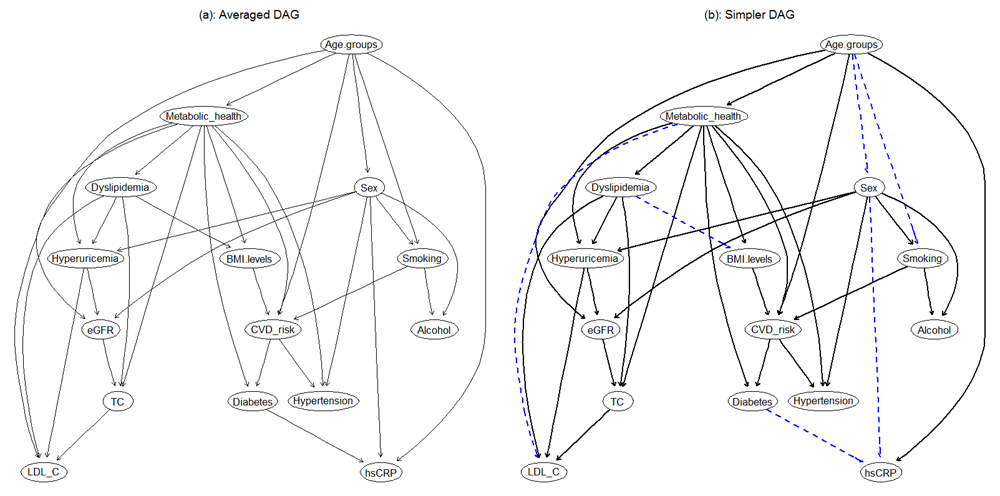

Supplement: Multimedia Appendix 1 [file medinform_v10i3e33026_app1.png]
